# Transition between Random and Periodic Electron Currents on a DNA Chain

**DOI:** 10.3390/ijms22147361

**Published:** 2021-07-08

**Authors:** Elham Faraji, Roberto Franzosi, Stefano Mancini, Marco Pettini

**Affiliations:** 1 Aix-Marseille Univ, Université de Toulon, CNRS, CPT, 13288 Marseille, France; elham.faraji@unicam.it; 2 CNRS Centre de Physique Théorique UMR7332, 13288 Marseille, France; 3 School of Science and Technology, University of Camerino, I-62032 Camerino, Italy; stefano.mancini@unicam.it; 4 INFN Sezione di Perugia, I-06123 Perugia, Italy; 5 QSTAR and INO-CNR, Largo Enrico Fermi 2, I-50125 Firenze, Italy; roberto.franzosi@ino.it

**Keywords:** DNA chains, Davydov model, electron current, Holstein-Fröhlich model, time dependent variational principle

## Abstract

By resorting to a model inspired to the standard Davydov and Holstein-Fröhlich models, in the present paper we study the motion of an electron along a chain of heavy particles modeling a sequence of nucleotides proper to a DNA fragment. Starting with a model Hamiltonian written in second quantization, we use the Time Dependent Variational Principle to work out the dynamical equations of the system. It can be found that, under the action of an external source of energy transferred to the electron, and according to the excitation site, the electron current can display either a broad frequency spectrum or a sharply peaked frequency spectrum. This sequence-dependent charge transfer phenomenology is suggestive of a potentially rich variety of electrodynamic interactions of DNA molecules under the action of electron excitation. This could imply the activation of interactions between DNA and transcription factors, or between DNA and external electromagnetic fields.

## 1. Introduction

A central question concerning the brain/mind activity and molecular dynamics in biological systems is whether in the warm, wet and noisy environment of living matter there are some processes for which quantum phenomena can play a relevant role. Some biological phenomena have been already ascribed to quantum effects, like in photosynthesis, bird orientation, and light response of opsins. In the search for relevant quantum effects in living matter, electrons are certainly good candidates, as is confirmed by the studies on the π-stacks of aromatic rings in proteins and in neuronal microtubules. Within this broad field of investigation, we focus on the behavior of electrons along DNA strands.

The possibility of charge transfer/transport along biomolecules, and notably along DNA, can have relevant biological consequences and has thus motivated several studies [1,2,3,4,5,6,7].

Following the quotations given in Ref. [7], electron transport on DNA can be involved in the action of DNA damage response enzymes, transcription factors, or polymerase co-factors, which are relevant processes in the cell [7,8]. For example, evidence has been given [7,9] that a DNA base excision repair enzyme enters the DNA repair process [10,11] through an electron transfer mechanism. Interestingly, electron transfer through damaged regions of DNA is markedly different with respect to electron transfer through healthy regions of DNA [10].

The aim of the present work is to investigate the spectral properties of an electron current generated along a segment of DNA. The reason for this study is two-fold, on the one side to investigate how DNA can respond to external electromagnetic excitations, on the other side with the perspective of a further investigation about the possible activation of selective attractive forces between selected sites of a DNA sequence and transcription factors.

Within a quantum mechanical framework, an electron transfer along some—essentially one dimensional—substrate is described by means of the probability current of the electron wave function ψ(t) which, in general, reads as

(1) $$\begin{array}{c} \vec{j}\left(\vec{x},t\right)=\frac{e\hbar }{2m_{e}i}\left(\psi^{*}\vec{\nabla }\psi -\psi \vec{\nabla }\psi^{*}\right) \end{array}$$

where *e* and $m_{e}$ are the electron charge and mass, respectively, and ℏ is the Planck’s constant; then, according to the D’Alambert equation $\partial^{\mu }\partial_{\mu }\vec{A}\left(t\right)=\mu_{0}e\vec{j}\left(\vec{x},t\right)$, where $\mu_{0}$ is the vacuum permeability, this current can generate an electromagnetic field of components

(2) $$\begin{array}{c} \vec{E}\left(t\right)=-\frac{\partial \vec{A}\left(t\right)}{\partial t}\\ \vec{B}\left(t\right)=\vec{\nabla }\times \vec{A}\left(t\right)\;. \end{array}$$

Then, the effect of the currents flowing along two macromolecules (1 and 2) can be that of generating an intermolecular force of electrodynamic kind given by the formula

(3) $${\vec{F}}_{12}\left(t\right)=-\frac{i_{1}\left(t\right)i_{2}\left(t\right)}{c^{2}}\oint \oint \frac{\left(d{\vec{l}}_{1}\cdot d{\vec{l}}_{2}\right){\vec{x}}_{12}}{|{\vec{x}}_{12}{|}^{3}}$$

where the double integral is a geometric form factor, and the currents $i_{1,2}\left(t\right)$ are given by the mean values

(4) $$i_{1,2}\left(t\right)=\frac{1}{l_{1,2}}\int_{0}^{l_{1,2}}{\vec{j}}_{1,2}\left(\vec{x},t\right)\;d\vec{x}$$

where $l_{1,2}$ stand for the linearized lengths of molecules 1 and 2, respectively.

According to the spectral properties of these currents, and in particular in case of the presence of co-resonances in their cross frequency spectrum ${\tilde{i}}_{1}^{*}\left(\omega \right){\tilde{i}}_{2}\left(\omega \right)$, the interaction force in Equation (3) could attain a strength of possible relevance in a biological context. Thus, the present work aims to contribute to an ongoing research field concerning the possible activation of intermolecular electrodynamic (resonant) forces [12,13,14].

The present work makes a first step in this direction by providing an investigation of the spectral properties of electron currents along DNA fragments.

## 2. Definition of the Model and Its Solution

There are several possible theoretical frameworks to model exciton propagation on a lattice, and the electron propagation on a lattice belongs to this class of phenomena. A paradigmatic model is the Haken–Strobl one [15] which describes different regimes of exciton–phonon interaction leading to the exciton damping through the scattering by lattice vibrations and thus describing the exciton motion by some kind of hopping process.

In the quantum biology literature, for example, the electronic energy transport to describe the coherent conveyance of electronic energy across chromophores of protein networks—via electrodynamic coupling of their transition electric dipole moments—is described by a tight-binding Hamiltonian for an interacting *N*-body system in the presence of a single excitation [16].

In the present work, in order to describe electronic motions along a DNA fragment, and from the perspective of the related electrodynamic interactions, we resort to a model partly borrowed from the standard Davydov and Holstein–Fröhlich models that have been originally introduced to account for electron–phonon interaction by explicitly also describing the dynamics of the underlying molecular lattice that, at room temperature, turns out to be essentially classical. Thus, to model the electrons moving along a given DNA sequence, the following Hamiltonian operator is assumed [17,18,19]

(5) $$\begin{array}{c} \hat{H}={\hat{H}}_{el}+{\hat{H}}_{ph}+{\hat{H}}_{int}, \end{array}$$

where the electronic and phononic parts of the Hamiltonian are given by

(6) $${\hat{H}}_{el}=\sum_{n=1}^{N}\left[E_{0}{\hat{B}}_{n}^{†}{\hat{B}}_{n}+\epsilon \langle {\hat{B}}_{n}^{†}{\hat{B}}_{n}\rangle {\hat{B}}_{n}^{†}{\hat{B}}_{n}+J_{n}\left({\hat{B}}_{n}^{†}{\hat{B}}_{n+1}+{\hat{B}}_{n}^{†}{\hat{B}}_{n-1}\right)\right],$$

(7) $${\hat{H}}_{ph}=\frac{1}{2}\sum_{n=1}^{N}\left[\frac{{\hat{p}}_{n}^{2}}{M_{n}}+\Omega_{n}{\left({\hat{u}}_{n+1}-{\hat{u}}_{n}\right)}^{2}+\frac{1}{2}\mu {\left({\hat{u}}_{n+1}-{\hat{u}}_{n}\right)}^{4}\right],$$

and the electron–phonon Hamiltonian reads as

(8) $${\hat{H}}_{int}=\sum_{n=1}^{N}\chi_{n}\left({\hat{u}}_{n+1}-{\hat{u}}_{n}\right){\hat{B}}_{n}^{†}{\hat{B}}_{n}.$$

As shown in the following, the introduction of the site-dependent electron–phonon coupling constant $\chi_{n}$ brings about a definitely richer phenomenology with respect to the site-independent case [20]. Here, we considered only a longitudinal sequence of nucleotides where ${\hat{B}}_{n}$ (${\hat{B}}_{n}^{†}$) is the electronic annihilation (creation) operator relative to the lattice sites n=1,2,...,N. The term $E_{0}{\hat{B}}_{n}^{†}{\hat{B}}_{n}$ accounts for the initial “bare” electron energy distributed on several lattice sites according to initial shape of the electron wavefunction. The new constant ϵ is the nonlinear electron–electron coupling energy due to the interaction of the moving electron along the DNA molecule with the electrons of the substrate of nucleotides and accounts for the Coulomb repulsion between the traveling electron and the charges localized on the nucleotides. The site-dependent parameter $J_{n}$ determines the strength of the nearest neighbor coupling energies of the electron tunneling across two neighboring nucleotides.

The vibronic part takes into account the longitudinal displacements of the nucleotides from their equilibrium positions, and each nucleotide at the *n*-th site along a DNA segment is described by the momentum and position operators, ${\hat{p}}_{n}$ and ${\hat{u}}_{n}$, by the mass $M_{n}$ and by the site-dependent $\Omega_{n}$, the spring parameter of two neighboring nucleotides. The parameter μ is the coupling constant of the nonlinear quartic term entailing the phonon–phonon interaction, of course absent in the harmonic approximation. The quartic term is introduced as a first (stable) term—beyond the harmonic approximation—in the power-law expansion around the minimum of typically nonlinear interparticle interaction potential (e.g., as is the case of Van der Waals potentials). The parameter $\chi_{n}$ is the site-dependent coupling parameter of the electron–lattice interaction.

Here we show how the quantum equations of motion for the Hamiltonian (5) can be derived using the time dependent variational principle (TDVP) in quantum mechanics. First, we work with the second of Davydov’s ansatz state vectors by assuming the following factorization

(9) $$\begin{array}{ccc} |\psi (t)\rangle & = & |\Psi \left(t\right)\rangle |\Phi \left(t\right)\rangle ;\;\;\;\;\;\;|\Psi \left(t\right)\rangle =\sum_{n}C_{n}\left(t\right){\hat{B}}_{n}^{†}{|0\rangle }_{el};\;\;\;\;\;\\ |\Phi (t)\rangle & = & e^{-\frac{i}{\hbar }\sum \left[\beta_{n}\left(t\right){\hat{p}}_{n}-\pi_{n}\left(t\right){\hat{u}}_{n}\right]}{|0\rangle }_{ph} \end{array}$$

normalized to unity by the condition $\langle \psi \left(t\right)|\psi \left(t\right)\rangle =\sum_{n}{\left|C_{n}\left(t\right)\right|}^{2}=1$, where $|C_{n}{\left(t\right)|}^{2}$ is the probability for finding the electron at the *n*-th site. The state vector |Ψ(t)〉 describes an electron given a single quantum excitation propagating along the DNA sequence of *N* nucleotides and |Φ(t)〉 describes the wave function from which the expectation values of ${\hat{u}}_{n}$ and ${\hat{p}}_{n}$ are obtained as

(10) $$\begin{array}{ccc} \langle \Phi |{\hat{u}}_{n}|\Phi \rangle & = & \beta_{n}\left(t\right),\\ \langle \Phi |{\hat{p}}_{n}|\Phi \rangle & = & \pi_{n}\left(t\right). \end{array}$$

According to TDVP, which is equivalent to the least action principle, we introduce a new wave function |ϕ(t)〉—in terms of |ψ(t)〉 in Equation (9)—by defining a time-dependent phase factor (S(t)∈R) so that

(11) $$\begin{array}{c} |\phi \left(t\right)\rangle =e^{iS(t)/\hbar }|\psi \left(t\right)\rangle , \end{array}$$

which implies the normalization 〈ϕ(t)|ϕ(t)〉=1. The wave function |ϕ(t)〉 satisfies the weaker form for the Schrödinger equation, $i\hbar \langle \phi \left(t\right)|\partial_{t}|\phi \left(t\right)\rangle =\langle \phi \left(t\right)|\hat{H}|\phi \left(t\right)\rangle$, giving

(12) $$\begin{array}{c} -\dot{S}\left(t\right)+i\hbar \langle \psi \left(t\right)|\partial_{t}|\psi \left(t\right)\rangle =\langle \psi \left(t\right)|\hat{H}|\psi \left(t\right)\rangle . \end{array}$$

Now, the TDVP reads as

(13) $$\begin{array}{c} \delta S\left(t\right)=0\;\;\;\;\;\;\;\mathrm{with}\;\;\;\;\;\;\;S\left(t\right)=\int_{0}^{t}L\left(t^{\prime }\right)dt^{\prime } \end{array}$$

where L(t) is the Lagrangian associated to the system

(14) $$\begin{array}{c} L\left(t\right)=i\hbar \langle \psi \left(t\right)|\partial_{t}|\psi \left(t\right)\rangle -\langle \psi \left(t\right)|\hat{H}|\psi \left(t\right)\rangle \;, \end{array}$$

hence and with Equation (9) we obtain

(15) $$\begin{array}{c} L=\sum_{n}\left\{i\hbar {\dot{C}}_{n}\left(t\right)C_{n}^{*}\left(t\right)+\frac{1}{2}\left(\pi_{n}\left(t\right){\dot{\beta }}_{n}\left(t\right)-{\dot{\pi }}_{n}\left(t\right)\beta_{n}\left(t\right)\right)-H\left(C_{n},C_{n}^{*},\beta_{n},\pi_{n}\right)\right\}, \end{array}$$

where

(16) $$\begin{array}{c} H\left(C_{n},C_{n}^{*},\beta_{n},\pi_{n}\right)=\langle \psi \left(t\right)|\hat{H}|\psi \left(t\right)\rangle . \end{array}$$

By requiring the fulfilment of the stationary action condition of Equation (13), we obtain

(17) $$\begin{array}{cc} \delta S(t) & =\sum_{n}\{i\hbar \left(-{\dot{C}}_{n}^{*}\left(t\right)\delta C_{n}\left(t\right)+{\dot{C}}_{n}\left(t\right)\delta C_{n}^{*}\left(t\right)\right)+{\dot{\beta }}_{n}\left(t\right)\delta \pi_{n}\left(t\right)-{\dot{\pi }}_{n}\left(t\right)\delta \beta_{n}\left(t\right)\\ & -\left(\partial_{C_{n}}H\right)\delta C_{n}-\left(\partial_{C_{n}^{*}}H\right)\delta C_{n}^{*}-\left(\partial_{\beta_{n}}H\right)\delta \beta_{n}-\left(\partial_{\pi_{n}}H\right)\delta \pi_{n}\}=0, \end{array}$$

and arrive at the dynamical equations

(18) $$\begin{array}{ccc} i\hbar {\dot{C}}_{n} & = & \partial_{C_{n}^{*}}H\\ {\dot{\beta }}_{n} & = & \partial_{\pi_{n}}H\\ {\dot{\pi }}_{n} & = & -\partial_{\beta_{n}}H\;. \end{array}$$

The expectation value of the Hamiltonian is

(19) $$\begin{array}{cc} \langle \psi |\hat{H}|\psi \rangle & =\sum_{n}[E_{0}|C_{n}{|}^{2}+\epsilon {\left|C_{n}\right|}^{4}+J_{n}\left(C_{n}^{*}C_{n+1}+C_{n+1}^{*}C_{n}\right)\\ & +\frac{1}{2}\left(\frac{1}{M_{n}}\pi_{n}^{2}+\Omega_{n}{\left(\beta_{n+1}-\beta_{n}\right)}^{2}+\frac{1}{2}\mu {\left(\beta_{n+1}-\beta_{n}\right)}^{4}\right)\\ & +\chi_{n}\left(\beta_{n+1}-\beta_{n}\right){\left|C_{n}\right|}^{2}]. \end{array}$$

Thus, from (18) and (19), we find the following explicit form of the equations of motion

(20) $$\begin{array}{ccc} i\hbar {\dot{C}}_{n} & = & \left(E_{0}+2\epsilon {\left|C_{n}\right|}^{2}+\chi_{n}\left(\beta_{n+1}-\beta_{n}\right)\right)C_{n}+J_{n}C_{n+1}+J_{n-1}C_{n-1},\\ M_{n}{\ddot{\beta }}_{n} & = & \Omega_{n}\beta_{n+1}+\Omega_{n-1}\beta_{n-1}-\Omega_{n-1}\beta_{n}-\Omega_{n}\beta_{n}+\chi_{n}|C_{n}{|}^{2}-\chi_{n-1}{\left|C_{n-1}\right|}^{2}\\ & + & \mu \left({\left(\beta_{n+1}-\beta_{n}\right)}^{3}-{\left(\beta_{n}-\beta_{n-1}\right)}^{3}\right). \end{array}$$

## 3. Physical Parameters

In order to choose meaningful physical coupling parameters of the Hamiltonian, we borrow from Refs. [21,22,23] the values of the interaction energy between an electron and each of all the four nucleotides (reported in Table 1). We assume that the moving electron—of initial energy $E_{0}$—moves along the sequence of nucleotides constituting a given segment of DNA by tunneling across square potential barriers of variable height and of width $a=3.4\;\overset{˚}{A}$, the average distance between two nearest neighboring nucleotides [17]. We introduce the transmission coefficients $T_{n,n+1}$ from the probability P(n→n±1) of tunneling from one potential well to the nearest one in order to set the electron hopping terms $J_{n}$ in (6)

(21) $$T_{n,n+1}={\left[1+\frac{E_{n+1}^{2}\sinh^{2}\left(\beta_{n+1}a\right)}{4E_{0}\left(E_{n+1}-E_{0}\right)}\right]}^{-1}\;,$$

where $\beta_{n+1}={\left[2m_{e}\left(E_{n+1}-E_{0}\right)/\hbar^{2}\right]}^{1/2}$, $m_{e}$ is the mass of electron and $E_{n+1}$ are the interaction energies between the moving electron and the local nucleotide. Then we assume

(22) $$\begin{array}{c} J_{n}=E_{0}T_{n,n+1}. \end{array}$$

For the Hamiltonian interaction (8) we can roughly estimate the electron–phonon coupling parameter $\chi_{n}$ as

(23) $$\begin{array}{c} \chi_{n}=dE/dx=\frac{E_{n+1}-E_{n}}{a}. \end{array}$$

Numerical simulations are performed by adopting dimensionless physical parameters in the dimensionless expressions of the Hamitonian (19) and of the dynamical Equation (20). These are found by rescaling time and lengths as $t=\omega^{-1}\tau$ and $\beta_{n}=Lb_{n}$, respectively, where $L=\sqrt{\hbar \omega^{-1}M_{n}^{-1}}$. The outcomes are

(24) $$\begin{array}{cc} \langle \psi |\hat{H}|\psi \rangle & =\sum_{n=1}^{N}[E^{\prime }|C_{n}{|}^{2}+\epsilon^{\prime }{\left|C_{n}\right|}^{4}+J_{n}^{\prime }\left(C_{n}^{*}C_{n+1}+C_{n+1}^{*}C_{n}\right)\\ \; & +\frac{1}{2}\left({\dot{b}}_{n}^{2}+\Omega_{n}^{\prime }{\left(b_{n+1}-b_{n}\right)}^{2}+\frac{1}{2}\mu^{\prime }{\left(b_{n+1}-b_{n}\right)}^{4}\right)\\ \; & +\chi_{n}^{\prime }\left(b_{n+1}-b_{n}\right){\left|C_{n}\right|}^{2}], \end{array}$$

and

(25) $$\begin{array}{ccc} i\frac{dC_{n}}{d\tau } & = & \left(E^{\prime }+2\epsilon^{\prime }{\left|C_{n}\right|}^{2}+\chi_{n}^{\prime }\left(b_{n+1}-b_{n}\right)\right)C_{n}+J_{n}^{\prime }C_{n+1}+J_{n-1}^{\prime }C_{n-1},\\ \frac{d^{2}b_{n}}{d\tau^{2}} & = & \Omega_{n}^{\prime }b_{n+1}+\Omega_{n-1}^{\prime }b_{n-1}-\Omega_{n-1}^{\prime }b_{n}-\Omega_{n}^{\prime }b_{n}+\chi_{n}^{\prime }|C_{n}{|}^{2}-\chi_{n-1}^{\prime }{\left|C_{n-1}\right|}^{2}\\ & + & \mu^{\prime }\left[{\left(b_{n+1}-b_{n}\right)}^{3}-{\left(b_{n}-b_{n-1}\right)}^{3}\right], \end{array}$$

where

(26) $$\begin{array}{ccc} E^{\prime } & = & \frac{E_{0}}{\hbar \omega };\;\;\;\;\;\;\;\epsilon^{\prime }=\frac{\epsilon }{\hbar \omega };\;\;\;\;\;\;\;J_{n}^{\prime }=\frac{J_{n}}{\hbar \omega };\;\;\;\;\;\;\;\\ \chi_{n}^{\prime } & = & \frac{\chi_{n}}{\sqrt{\hbar M_{n}\omega^{3}}};\;\;\;\;\;\Omega_{n}^{\prime }=\frac{\Omega_{n}}{M_{n}\omega^{2}};\;\;\;\;\;\;\mu^{\prime }=\frac{\mu \hbar }{M_{n}^{2}\omega^{3}}\;. \end{array}$$

In our simulations we resort to the known sound speed $V=a{\left(\Omega_{n}/M_{n}\right)}^{1/2}$ on DNA; we borrow the value V=1.69 km/s from [24] and take it as an average constant (neglecting small local variations due to the different masses of the nucleotides). Thus, we obtain the constant dimensionless parameter $\Omega^{\prime }=V^{2}/a^{2}\omega^{2}$ from (26). In re-writing the dynamical equations we introduce the variables

(27) $$\begin{array}{c} q_{n}=\frac{C_{n}+C_{n}^{*}}{\sqrt{2}},\;p_{n}=\frac{C_{n}-C_{n}^{*}}{i\sqrt{2}}\;, \end{array}$$

so that Equation (25) become

(28) $$\begin{array}{cc} {\dot{q}}_{n} & =\left[E^{\prime }+\frac{\epsilon^{\prime }}{2}\left(q_{n}^{2}+p_{n}^{2}\right)+\chi_{n}^{\prime }\left(b_{n+1}-b_{n}\right)\right]p_{n}+J_{n}^{\prime }p_{n+1}+J_{n-1}^{\prime }p_{n-1}, \end{array}$$

(29) $$\begin{array}{cc} {\dot{p}}_{n} & =-\left[E^{\prime }+\frac{\epsilon^{\prime }}{2}\left(q_{n}^{2}+p_{n}^{2}\right)+\chi_{n}^{\prime }\left(b_{n+1}-b_{n}\right)\right]q_{n}+J_{n}^{\prime }q_{n+1}+J_{n-1}^{\prime }q_{n-1}], \end{array}$$

(30) $$\begin{array}{cc} {\ddot{b}}_{n} & =\Omega^{\prime }\left(b_{n+1}+b_{n-1}-2b_{n}\right)+\frac{1}{2}\left(\chi_{n}^{\prime }\left(q_{n}^{2}+p_{n}^{2}\right)-\chi_{n-1}^{\prime }\left(q_{n-1}^{2}+p_{n-1}^{2}\right)\right)\\ \; & +\mu^{\prime }\left[{\left(b_{n+1}-b_{n}\right)}^{3}-{\left(b_{n}-b_{n-1}\right)}^{3}\right]\\ \; & =B_{n}\left[b\left(t\right),q\left(t\right),p\left(t\right)\right]\;. \end{array}$$

where the equation for ${\ddot{b}}_{n}$ can be also written as

(31) $$\begin{array}{ccc} {\dot{b}}_{n} & = & \pi_{n}\\ {\dot{\pi }}_{n} & = & B_{n}\left[b\left(t\right),q\left(t\right),p\left(t\right)\right]\;. \end{array}$$

Finally, by combining a leap-frog scheme for (31) and a finite differences scheme for ${\dot{q}}_{n}$ and ${\dot{p}}_{n}$, Equations (28)–(30) are rewritten in a form suitable for numerical solution, that is

(32) $$\begin{array}{ccc} q_{n}\left(t+\Delta t\right) & = & q_{n}\left(t\right)+\Delta t\;Q_{n}\left[b\left(t\right),q\left(t\right),p\left(t\right)\right],\\ p_{n}\left(t+\Delta t\right) & = & p_{n}\left(t\right)+\Delta t\;P_{n}[b\left(t\right),q\left(t\right),p\left(t\right)],\\ b_{n}\left(t+\Delta t\right) & = & b_{n}\left(t\right)+\Delta t\;\pi_{n}\left(t\right),\\ \pi_{n}\left(t+\Delta t\right) & = & \pi_{n}\left(t\right)+\Delta t\;B_{n}\left[b\left(t+\Delta t\right),q\left(t+\Delta t\right),p\left(t+\Delta t\right)\right], \end{array}$$

where $Q_{n}\left[b\left(t\right),q\left(t\right),p\left(t\right)\right]$ and $P_{n}\left[b\left(t\right),q\left(t\right),p\left(t\right)\right]$ are the r.h.s. of Equations (28) and (29), respectively. The integration scheme for $b_{n}\left(t\right)$ and $\pi_{n}\left(t\right)$ is a symplectic one, meaning that all the Poincaré invariants of the associated Hamiltonian flow are conserved, among these invariants there is energy. The simple leap-frog scheme cannot be applied to the equations for ${\dot{q}}_{n}\left(t\right)$ and ${\dot{p}}_{n}\left(t\right)$ because the r.h.s. of the equations for ${\dot{q}}_{n}\left(t\right)$ explicitly depend on $q_{n}\left(t\right)$ and $b_{n}\left(t\right)$; therefore, the first two equations in (32) are integrated with an Euler predictor–corrector to give

(33) $$\begin{array}{ccc} \;\;\;\;\;q_{n}^{\left(0\right)}\left(t+\Delta t\right) & = & q_{n}\left(t\right)+\Delta t\;Q_{n}\left[b\left(t\right),q\left(t\right),p\left(t\right)\right],\\ p_{n}^{\left(0\right)}\left(t+\Delta t\right) & = & p_{n}\left(t\right)+\Delta t\;P_{n}\left[b\left(t\right),q\left(t\right),p\left(t\right)\right],\\ q_{n}^{\left(1\right)}\left(t+\Delta t\right) & = & q_{n}\left(t\right)+\frac{\Delta t}{2}\left\{Q_{n}\left[b\left(t\right),q\left(t\right),p\left(t\right)\right]+Q_{n}\left[b\left(t\right),q^{\left(0\right)}\left(t+\Delta t\right),p^{\left(0\right)}\left(t+\Delta t\right)\right]\right\},\\ p_{n}^{\left(1\right)}\left(t+\Delta t\right) & = & p_{n}\left(t\right)+\frac{\Delta t}{2}\left\{P_{n}\left[b\left(t\right),q\left(t\right),p\left(t\right)\right]+P_{n}\left[b\left(t\right),q^{\left(0\right)}\left(t+\Delta t\right)\right),p^{\left(0\right)}\left(t+\Delta t\right)]\right\},\\ b_{n}\left(t+\Delta t\right) & = & b_{n}\left(t\right)+\Delta t\;\pi_{n}\left(t\right),\\ \pi_{n}\left(t+\Delta t\right) & = & \pi_{n}\left(t\right)+\Delta t\;B_{n}\left[b\left(t+\Delta t\right),q^{\left(1\right)}\left(t+\Delta t\right),p^{\left(1\right)}\left(t+\Delta t\right)\right], \end{array}$$

so that, thanks to fact that half of the set of the dynamical Equation (32) is integrated by means of a symplectic algorithm, and half of the equations are integrated by means of the Euler predictor–corrector, it turns out that by adopting sufficiently small integration time steps Δt the total energy is very well conserved without any drift, just with zero-mean fluctuations around a given value fixed by the initial conditions. Regarding initial conditions, independently of the specific physical excitation mechanism, we assume an electron wavefunction described by the amplitudes $C_{n}\left(t=0\right)$ centered at the excitation site $n=n_{0}$ and distributed at time t=0 [17] as

(34) $$\begin{array}{ccc} C_{n}\left(t=0\right) & = & \frac{1}{\sqrt{8\sigma_{0}}}\mathrm{sech}\left(\frac{n-n_{0}}{4\sigma_{0}}\right) \end{array}$$

where $\sigma_{0}$ specifies the width of the function. Periodic boundary conditions have been used.

Regarding the initial conditions of the phonon part of the system, we assume that the components of the DNA fragment under consideration are initially thermalized at the room temperature T=310K. At thermal equilibrium, the average kinetic and potential energy per degree of freedom are equal, therefore at t=0 the displacements and the associated velocities were initialized with random values of zero mean and according to the following prescription

(35) $$\langle |b_{n}{\left(0\right)|\rangle }_{n}=\sqrt{\frac{k_{B}T}{\hbar \omega \Omega^{\prime }}};\;\;\;\;\;\;\;\;\;\;\;\;\;\;\;\;\;\langle |{\dot{b}}_{n}\left(0\right){|\rangle }_{n}=\sqrt{\frac{k_{B}T}{\hbar \omega }}.$$

expressed in dimensionless form. Periodic boundary conditions have been used also for the phonon part of the system.

## 4. Numerical Results

In numerical simulations, we adopted an integration time step $\Delta t=5\times 10^{-6}$ (dimensionless units) entailing a very good energy conservation with typical fluctuations of relative amplitude $\Delta E/E≃10^{-6}$.

In what follows, we report the results obtained for two sequences of N=66 and N=398 nucleotides, respectively, for different initial electron energies $E_{0}$, and for initial excitation sites $n_{0}$ entering the initial wavefunction shape (34).

As described in the Introduction, the physical quantity of interest in what follows is the Fourier spectrum of the electron current activated on a segment of DNA. The average electron current is derived from the standard probability current $j(x_{i},t)$ associated with the wave function |ψ(t)〉 in (9), which, in the discretized version along the chain of nucleotides and thus ready for its numerical computation, reads as

(36) $$\begin{array}{c} j\left(x_{i},t\right)=\frac{e\hbar }{2m_{e}i}\left(\psi^{*}\left(x_{i},t\right)\frac{\psi \left(x_{i+1},t\right)-\psi \left(x_{i-1},t\right)}{2a}-\psi \left(x_{i},t\right)\frac{\psi^{*}\left(x_{i+1},t\right)-\psi^{*}\left(x_{i-1},t\right)}{2a}\right) \end{array}$$

and hence the average current

(37) $$i_{av}\left(t\right)=\frac{1}{l}\int_{0}^{l}j\left(x,t\right)dx=\frac{1}{Na}\sum_{i=1}^{N}j\left(x_{i},t\right)a\;.$$

**Figure 1:**
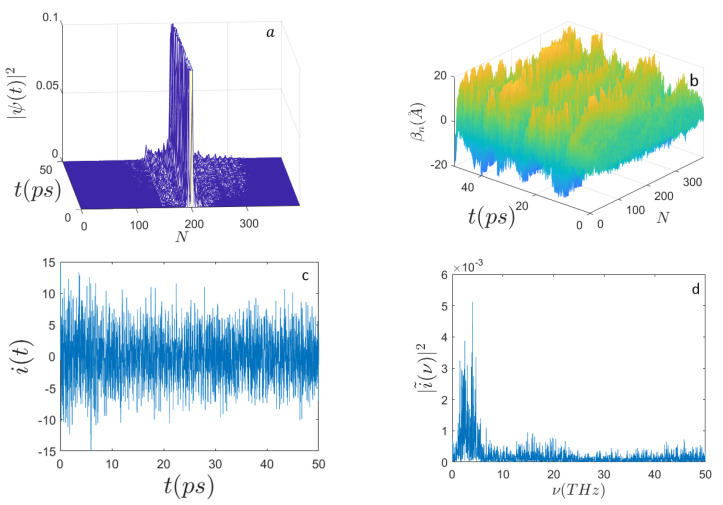
Time evolution of (**a**) the electron probability amplitude ${\left|\Psi \left(t\right)\right|}^{2}$; (**b**) the average displacements $\beta_{n}\left(\overset{˚}{A}\right)$; (**c**) the electron current i(t) (expressed in μbiot); (**d**) the frequency spectrum $|\tilde{i}{\left(\nu \right)|}^{2}$ along the sequence of N=398 nucleotides. Initial conditions: $T=310^{\circ }$ K, $n_{0}=N/2$, $E^{\prime }=100$, $\epsilon^{\prime }=5$, $\Omega^{\prime }=0.25$, $\mu^{\prime }=0.5$, $\sigma_{0}=0.1$, and the site-dependent parameters $J_{n}^{\prime }$, and $\chi_{n}^{\prime }$ corresponding to $E_{0}=0.658$ eV, ϵ=0.0329 eV, $\Omega_{n}=V^{2}M_{n}/a^{2}$, $J_{n}$, and $\chi_{n}$ in Equations (22) and (23), respectively. Initial electron excitation peaked around the site $n_{0}=N/2$.

**Figure 2:**
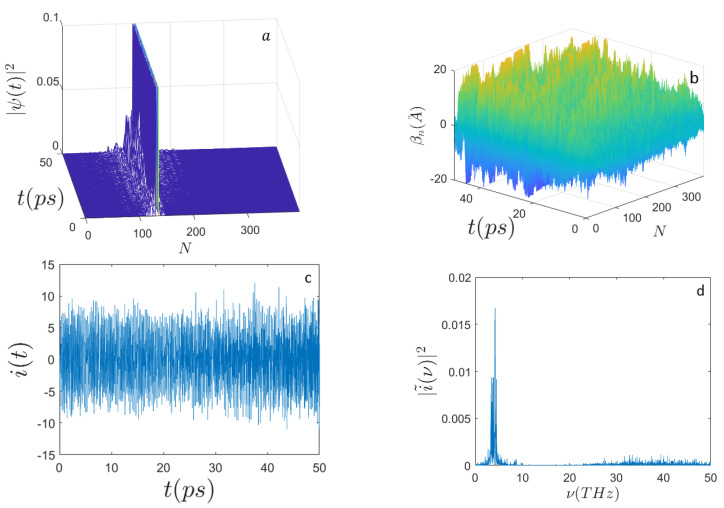
Time evolution of (**a**) the electron probability amplitude ${\left|\Psi \left(t\right)\right|}^{2}$; (**b**) the average displacements $\beta_{n}\left(\overset{˚}{A}\right)$; (**c**) the electron current i(t) (expressed in μbiot); (**d**) the frequency spectrum $|\tilde{i}{\left(\nu \right)|}^{2}$ along the sequence of N=398 nucleotides. Initial conditions and parameters are the same as those for Figure 1 but the initial electron excitation peaks around the site $n_{0}=N/3$.

**Figure 3:**
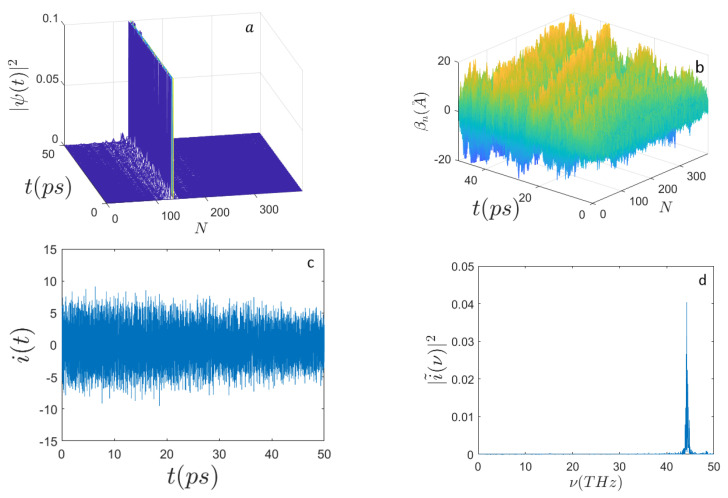
Time evolution of (**a**) the electron probability amplitude ${\left|\Psi \left(t\right)\right|}^{2}$; (**b**) the average displacements $\beta_{n}\left(\overset{˚}{A}\right)$; (**c**) the electron current i(t) (expressed in μbiot); (**d**) the frequency spectrum $|\tilde{i}{\left(\nu \right)|}^{2}$ along the sequence of N=398 nucleotides, with the initial condition $E_{0}=0.79$ eV ($E^{\prime }=120$) and the initial electron excitation peaked around the site $n_{0}=N/3$. The other parameters are the same as those in Figure 1.

**Figure 4:**
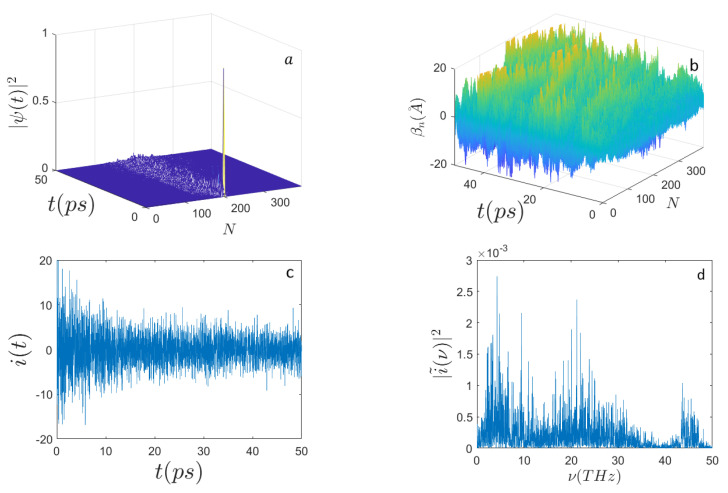
Time evolution of (**a**) the electron probability amplitude ${\left|\Psi \left(t\right)\right|}^{2}$; (**b**) the average displacements $\beta_{n}\left(\overset{˚}{A}\right)$; (**c**) the electron current i(t) (expressed in μbiot); (**d**) the frequency spectrum $|\tilde{i}{\left(\nu \right)|}^{2}$ along the sequence of N=398 nucleotides with the initial condition $E_{0}=0.46$ eV ($E^{\prime }=70$). The initial electron excitation peaks around the site $n_{0}=N/2$. The other parameters are the same as those in Figure 1.

In Figure 1, Figure 2, Figure 3 and Figure 4 the outcomes are reported of the simulations performed for a DNA sequence coding for a subunit of the human haemoglobin molecule (HBB) consisting of N=398 nucleotides (see Appendix A). Figure 1 and Figure 2 display the behavior of the system when $E_{0}=0.658$ eV and for $n_{0}=N/2$ and $n_{0}=N/3$, respectively. Remarkably, the mean electron current is alternate in that it takes positive and negative values; however, the corresponding Fourier power spectra appear very different according to the initial excitation site. In fact, for $n_{0}=N/3$ it is well evident that the spectrum $|\tilde{i}{\left(\nu \right)|}^{2}$ peaks around the frequency ν≃5 THz, whereas for $n_{0}=N/2$ the spectrum appears much broader and noisy.

Then, by comparing the results obtained keeping the initial excitation around the site $n_{0}=N/3$ but increasing the energy to $E_{0}=0.79$ eV, an interesting and to some extent surprising result is found: the power spectrum of the current becomes sharper and peaks around ν≃44 THz, a much higher frequency indeed. This is shown in Figure 3.

On the other hand, with the initial excitation again localized around the site $n_{0}=N/2$ and with a lower initial activation energy, $E_{0}=0.46$ eV, Figure 4 shows that the electron wave function quickly spreads over the whole chain of nucleotides and the electron current power spectrum broadens with respect to the one found for $E_{0}=0.658$ eV. Of course, the parameter space of the system is very large and thus its systematic investigation is practically unfeasible. Nevertheless, the above reported results outline the existence of a richer phenomenology with respect to the excitation of just Davydov electro-solitons. This could be of interest in view of modeling specific processes involving electrodynamic interactions of DNA with other biomolecules or with external electromagnetic fields.

Panel (b) of the different figures shows the time evolution of random initial conditions for the displacements of the underlying sequences of masses modeling nucleotides of the DNA. According to the prescriptions of Equation (35), the random initial displacements and velocities are made at thermal equilibrium at $310^{\circ }$ K.

**Figure 5:**
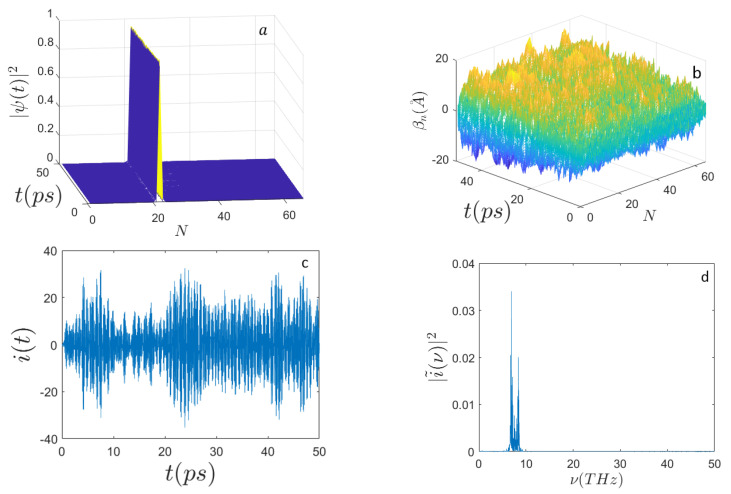
Time evolution of (**a**) the electron probability amplitude ${\left|\Psi \left(t\right)\right|}^{2}$; (**b**) the average displacements $\beta_{n}\left(\overset{˚}{A}\right)$; (**c**) the electron current i(t) (expressed in μbiot); (**d**) the frequency spectrum $|\tilde{i}{\left(\nu \right)|}^{2}$ along the sequence of N=66 nucleotides with the initial conditions $E_{0}=0.2$ eV ($E^{\prime }=30$) and the initial electron excitation peaked around the site $n_{0}=N/3$. The other parameters are the same of Figure 1.

**Figure 6:**
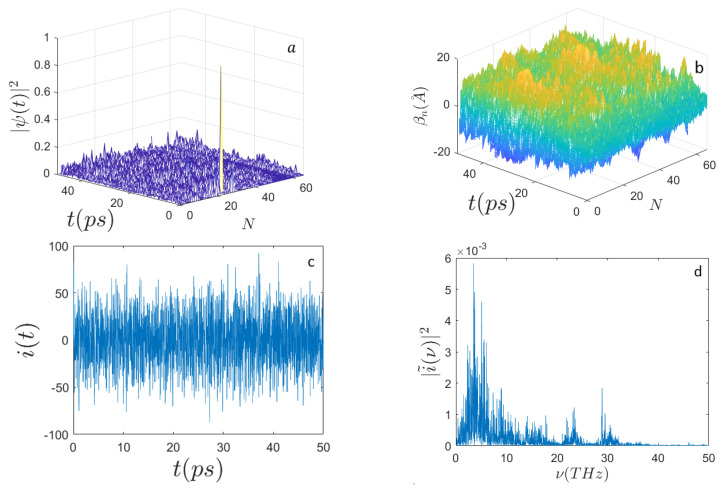
Time evolution of (**a**) the electron probability amplitude ${\left|\Psi \left(t\right)\right|}^{2}$; (**b**) the average displacements $\beta_{n}\left(\overset{˚}{A}\right)$; (**c**) the electron current i(t) (expressed in μbiot); (**d**) the frequency spectrum $|\tilde{i}{\left(\nu \right)|}^{2}$ along the sequence of N=66 nucleotides with the initial conditions $E_{0}=0.79$ eV ($E^{\prime }=120$) and the initial electron excitation peaked around the site $n_{0}=N/3$. The other parameters are the same as those in Figure 1.

**Figure 7:**
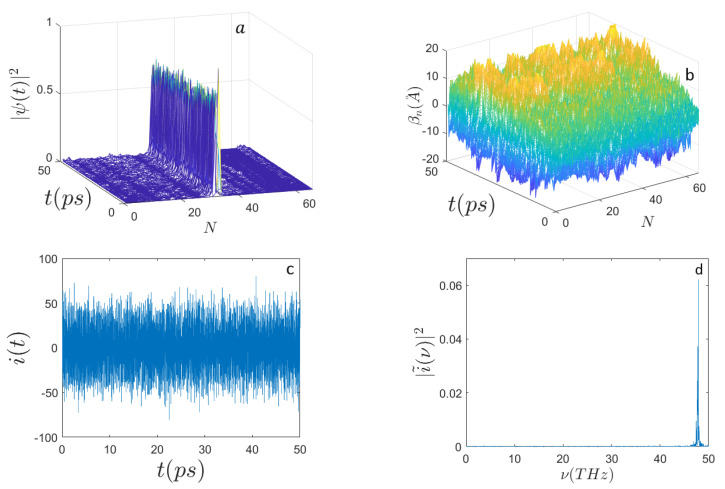
Time evolution of (**a**) the electron probability amplitude ${\left|\Psi \left(t\right)\right|}^{2}$; (**b**) the average displacements $\beta_{n}\left(\overset{˚}{A}\right)$; (**c**) the electron current i(t) (expressed in μbiot); (**d**) the frequency spectrum $|\tilde{i}{\left(\nu \right)|}^{2}$ along the sequence of N=66 nucleotides with the initial conditions $E_{0}=0.79$ eV ($E^{\prime }=120$) and the initial electron excitation peaking around the site $n_{0}=N/2$. The other parameters are the same as those in Figure 1.

**Figure 8:**
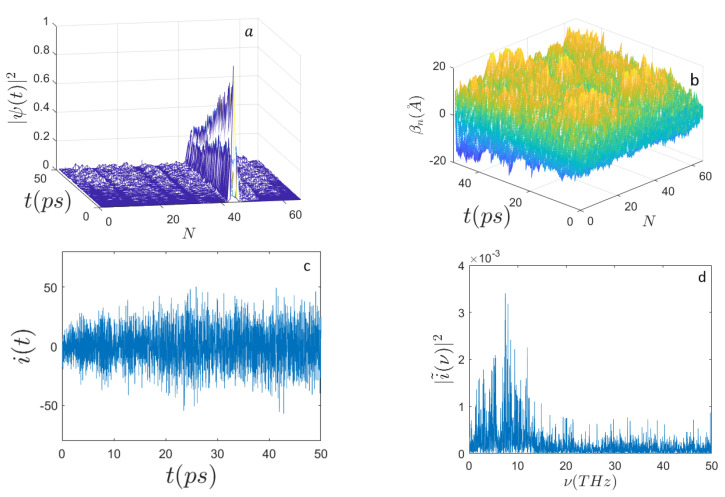
Time evolution of (**a**) the electron probability amplitude ${\left|\Psi \left(t\right)\right|}^{2}$; (**b**) the average displacements $\beta_{n}\left(\overset{˚}{A}\right)$; (**c**) the electron current i(t) (expressed in μbiot); (**d**) the frequency spectrum $|\tilde{i}{\left(\nu \right)|}^{2}$ along the sequence of N=66 nucleotides with the initial conditions $E_{0}=0.9$ eV ($E^{\prime }=136.7$) and the initial electron excitation peaking around the site $n_{0}=2N/3$. The other parameters are the same as those in Figure 1.

In Figure 5, Figure 6, Figure 7 and Figure 8 the results are reported as numerical simulations obtained for a shorter sequence of N=66 nucleotides containing the GAATTC recognition site of the restriction endonuclease enzyme EcoRI [25,26] (see Appendix B). These results confirm the richness of the phenomenology previously observed for the longer DNA sequence. The spatial distribution of the probability ${\left|\Psi \left(x,t\right)\right|}^{2}$ in Figure 5 where $E_{0}=0.2$ eV and $n_{0}=N/3$ seems completely frozen in time even though some low amplitude ripple exists which entail a non-vanishing electron current; the power spectrum $|\tilde{i}{\left(\nu \right)|}^{2}$ shows some peaks concentrated in the frequency interval 5–10 THz. By increasing the initial excitation energy of the electron above 0.6 eV, and keeping the excitation centered at the same site $n_{0}=N/3$, we can see in Figure 6 a quick and complete spreading of the electron wave function ${\left|\Psi \left(x,t\right)\right|}^{2}$ and, correspondingly, a broad and noisy power spectrum of the electron current. A further increase in the electron excitation energy at $E_{0}=0.79$ eV, with the initial wavefunction centered at the site $n_{0}=N/2$, brings about a well evident ripple of ${\left|\Psi \left(x,t\right)\right|}^{2}$ around its initial peak, as can be seen in Figure 7. The associated electron current power spectrum turns out to peak drastically around 48 THz. Then, considering the initial electron energy at $E_{0}=0.9$ eV and displacing the initial excitation around the site $n_{0}=2N/3$ it is observed that the peak value of the electron wavefunction decreases much faster than in the previous case ($n_{0}=N/2$) and also the power spectrum of the current is very different from the previous case, displaying a broad and noisy pattern, as can be seen in Figure 8.

A comment about the possible biological significance of the energies adopted for the simulations is in order. The electron excitation values of 0.2, 0.46, and 0.6 eV correspond to one, two and three DNA phosphodiester bonds, respectively, and are the same of those considered in [27] where an analysis was carried out for the same restriction enzyme DNA target sequence considered in the present work.

**Figure 9:**
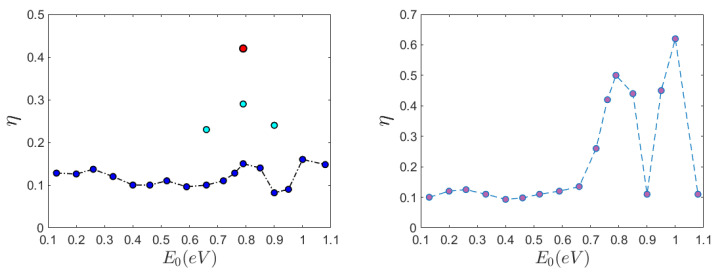
The normalized spectral entropy η versus the initial energy $E_{0}$ for a DNA protein. The parameters are the same as those reported for Figure 1. The left panel refers to N=398 bp where the dark blue circles correspond to the initial electron excitation peaking around the site $n_{0}=N/2$, the light blue circles correspond to $n_{0}=N/3$, and the red circle correspond to $n_{0}=2N/3$. The right panel refers to N=66 bp and the initial electron excitation peaks around the site $n_{0}=N/2$.

A complementary characterization of the electron current power spectra can be performed by means of spectral entropy. This is defined *à la* Shannon as follows

(38) $$S\left(t\right)=-\sum_{m=1}^{M/2}w_{m}\left(t\right)\ln w_{m}\left(t\right);\;\;\;\;\;\;\;\;w_{m}\left(t\right)=\frac{E_{m}\left(t\right)}{E_{T}\left(t\right)}$$

the weights $w_{m}\left(t\right)$ are normalized by

(39) $$E_{T}\left(t\right)=\sum_{m=1}^{M/2}E_{m}\left(t\right);\;\;\;\;\;\;\;\;E_{m}\left(t\right)=\nu_{m}^{2}{\left|\tilde{i}\left(\nu_{m}\right)\right|}^{2}$$

where $\tilde{i}\left(\nu_{m}\right)$ indicates the Fourier transform of the electron current. The frequencies are $\nu_{m}=m/T=m/\left(M\Delta T\right)$ where *T* is the length of time window which is Fourier analyzed, ΔT is a sampling time such that T=MΔT. As the input signals are real numbers and the Fourier spectra are computed through DFT algorithm we ignore the mirror part of the spectra thus ignoring the second half of the FFT. Then, the spectral entropy is normalized to give

(40) $$\eta \left(t\right)=\frac{S_{max}\left(t\right)-S\left(t\right)}{S_{max}\left(t\right)}$$

so that it is η=1 when the power spectrum of the electron current is monochromatic, and η=0 for a flat spectrum such that $S\left(t\right)=S_{max}\left(t\right)$. Figure 9 shows the normalized entropy η of the electrons versus the initial energy $E_{0}$. In panel (a) η versus $E_{0}$ is reported for the longer DNA segment under different initial conditions: for $n_{0}=N/2$ the normalized entropy η takes values approximately in the interval 0.1–0.15 meaning that the corresponding power spectra are broad and noisy; for initial excitation site $n_{0}=N/3$ it can be found η=0.45 at $E_{0}=0.79$ eV and we recover what has been already displayed by Figure 3, that is, the power spectrum is narrow in frequency; intermediate values of η can be found when the excitation site is $n_{0}=2N/3$ at energies $E_{0}=0.66,0.79,0.9$ eV.

In panel (b) η versus $E_{0}$ is reported for the shorter DNA segment and synoptically shows the nontrivial appearance of some kind of, loosely speaking, “resonances” in which η displays some bumps in its $E_{0}$-pattern which correspond to a significant narrowing of the electron current spectra, and thus to a less noisy and more coherent behavior of the electron current.

## 5. Concluding Remarks

The novelty of the model investigated in the present work concerns the introduction of site-dependent electron–phonon coupling constants. The sequence of values of these coupling constants follows the sequence of nucleotides along a given DNA segment. Moreover, the sequence of probabilities of electron jumping from one site to the next one depends on the specific sequence of nucleotides. In so doing, a richer phenomenology is found with respect to a similar model recently investigated [20]. Instead of observing the propagation of a standard Davydov electro-soliton, depending on the initial excitation energy, we observed localized periodic motions of the electrons—giving rise to a narrow frequency spectrum of the electron current—or, depending on the initial excitation site, to more extended motions associated with a broad noisy frequency spectrum. In both cases, the relevant spectral range belongs to the THz frequency domain. A qualitatively similar phenomenology was found by tackling two different DNA molecules, a subunit of the haemoglobin molecule (HBB) and an oligonucleotide with a specific recognition site of a restriction enzyme. Our findings suggest that the activation of periodic currents on specific sites could be at the origin of attractive forces between the DNA and a specific effector (transcription factor, enhancer, inhibitor, and so on). The prospective developments of the present work thus concern a new investigation/explanation of the physical grounds of the Resonant Recognition Model [21,22] by looking for co-resonances in the current frequency spectra of biochemical reaction partners. Moreover, electromagnetic signaling from electronic currents flowing along a DNA strand, and DNA response to externally applied electromagnetic fields could be further investigated in the light of the approach proposed in the present work.

Finally, further developments of the present model, aimed at describing co-resonance-activated intermolecular interactions, will have to take into account the role of hydration layers of the interacting bio-molecules. In fact, as put forward in [12], hydration layers made of spatially ordered water dipole moments can deeply affect the strength of the intermolecular electrodynamic interactions and the existence and relevance of these hydration layers is experimentally proved for DNA molecules [28,29] and for proteins [30].

## Figures and Tables

**Table 1:** Electron–Ion interaction potential (EIIP) values for nucleotides adenine (A), thymine (T), guanine (G), and cytosine (C). From Refs. [21,22].

Nucleotide	EIIP Ry	EIIP eV	Nucleotide	EIIP Ry	EIIP eV
A	0.1260	1.7143	T	0.1335	1.8164
G	0.0806	1.0966	C	0.1340	1.8232

## Data Availability

Data are available upon request.

## Appendix A

CGGCTGTCATCACTTAGACCTCACCCTGTGGAGCCACACCCTAGGGTT

GGCCAATCTACTCCCAGGAGCAGGG AGGGCAGGAGCCAGGGCTGGGCATAAA

AGTCAGGGCAGAGCCATCTATTGCTTACATTTGCTTCTGACACAACTGTGTTCACT

AGCAACCTCAAACAGACACCATGGTGCATCTGACTCCTGTGGAGAAGTCTGCCGT

TACTGCCCTGTGGGGCAAGGTGAACGTGGATGAAGTTGGTGGTGAGGCCCTGGG

CAGGTTGGTATCAAGGTTACAAGACAGGTTTAAGGAGACCAATAGAAACTGGGCAT

GTGGAGACAGAGAAGACTCTTGGGTTTCTGATAGGCACTGACTCTCTCTGCCTATT

GGTCTATTTTCCCACCCTTAG

Gene sequence (398 bp) coding for one of the β subunits of the haemoglobin molecule (HBB)

## Appendix B

ATGGCTAATGACCGAGAATAGGGATCCGAATTCAATATTGGTACCTA

CGGGCTTTGCGCTCGTATC

Sequence of an oligonucleotide (66 bp) containing a cleavage subsequence of the EcoRI enzyme
